# Computer-assisted cannulated screw internal fixation versus conventional cannulated screw internal fixation for femoral neck fractures: a systematic review and meta-analysis

**DOI:** 10.1186/s13018-021-02806-7

**Published:** 2021-11-22

**Authors:** Qing-hao Cheng, Peng-biao Li, Ting-ting Lu, Shi-fang Guo, Wen-fei Di, Ke-hu Yang, Yao-wen Qian

**Affiliations:** 1 Gansu Provincial Hospital, 204 Donggang West Road, Chengguan District, Lanzhou, 730000 China; 2 Institution of Clinical Research and Evidence-Based Medicine, The Gansu Provincial Hospital, Lanzhou, China; 3 Evidence-Based Medicine Center, School of Basic Medical Sciences, Lanzhou University, No. 222, Tianshui South Road, Chengguan District, Lanzhou, 730000 China; 4 Key Laboratory of Evidence-Based Medicine and Knowledge Translation of Gansu Province, Lanzhou, China; 5 School of Basic Medical Sciences, Ningxia Medical University, Yinchuan, China

## Abstract

**Objective:**

To compare the effects between computer-assisted and traditional cannulated screw internal fixation on treating femoral neck fracture.

**Methods:**

The search was conducted in Embase, Pubmed, Web of Science, Cochrane Library, China National Knowledge Infrastructure (CNKI) and Wanfang Database from the beginning to August 2020. RevMan5.4 software, which was provided by the International Cochrane Group, was used for the meta-analysis comparing the differences in operation time, intraoperative bleeding volume, fluoroscopy frequency, fracture healing time, total drilling times, Harris score, fracture healing rate, and femoral head necrosis rate between computer-assisted and traditional methods groups.

**Results:**

A total of 1028 patients were included in 16 studies. Primary outcome indicators: Compared with the traditional method group, the computer-assisted group had less operative time (2RCTs, *P* < 0.00001; 8 non-RCTs, *P* = 0.009; Overall, *P* < 0.00001), intraoperative bleeding (1 RCTs, *P* < 0.00001; 9non-RCTs, *P* < 0.00001; Overall, *P* < 0.00001), femoral head necrosis rate (1 RCT, *P* = 0.11;7 non-RCTs, *P* = 0.09; Overall, *P* = 0.02) and higher Harris scores (1 RCT, *P* < 0.0001; 9 non-RCTs, *P* = 0.0002; Overall, *P* < 0.0001), and there were no significant differences in fracture healing rate between the two groups (5 non-RCTs, *P* = 0.17). Secondary outcomes indicators: The computer-assisted group had a lower frequency of intraoperative fluoroscopy and total number of drills compared with the traditional method group, while there was no significant difference in fracture healing time.

**Conclusion:**

Compared with the traditional hollow screw internal fixation on the treatment of femoral neck fracture, computer-assisted percutaneous cannulated screw fixation can shorten the operation time and improve the operation efficiency and reduce the X-ray injury of medical staff and help patients obtain a better prognosis. Therefore, computer-assisted percutaneous cannulated screw fixation is a better choice for the treatment of femoral neck fracture.

*Study registration* PROSPERO registration number CRD42020214493.

**Supplementary Information:**

The online version contains supplementary material available at 10.1186/s13018-021-02806-7.

## Introduction

The femoral neck fracture is the most common hip fracture, which often occurs in elderly patients with osteoporosis, and it accounts for 3.58% and 54% of systemic fractures and hip fractures [1]. The incidence of young people is relatively low. Femoral neck fractures tend to be caused by high-energy injuries, which account for only 2 to 3% of all femoral neck fractures [1–3]. Therefore, femoral neck fracture has become a thorny problem in clinical treatment. Currently, surgery is the first choice of therapy for femoral neck fractures. For femoral neck fractures without displacement or where satisfactory reduction has been obtained, the most common treatment is an internal fixation with closed reduction hollow screws [4, 5]. Several studies have shown that an inverted equilateral triangle is formed between a screw and another closed one, effectively preventing the femur's subtrochanteric fracture and providing better biomechanical stability [6, 7]. At the same time, accurate screw placement can increase the stability of internal fixation of a femoral neck fracture and reduce the risk of nonunion [8, 9].

However, traditional cannulated screw internal fixation has many disadvantages. For example, the instability of the direction of the guide during the drilling process can lead to bone damage caused by repeated drilling. It can also cause the dislocation of the screws to penetrate the lateral cortex [2]. Surgery by traditional methods requires continuous fluoroscopy by an experienced surgeon to obtain a more accurate screw location. Still, it is also difficult to ensure that the screw is placed in the best position during the procedure [10]. These factors directly or indirectly lead to postoperative complications, such as fracture nonunion, femoral head necrosis, failure of internal fixation, etc., and affect the functional prognosis [11]. Besides, frequent intraoperative fluoroscopy also increases radiation exposure of medical staff and patients when determining the location of guidewires and screws [2]. With the advancement of medical technology and the increasing demand for minimally invasive surgical treatment, traditional surgery cannot meet the needs of the times. The emergence of the orthopedic robot not only makes up for the shortcomings of traditional surgery but also provides functions such as surgical navigation, planning simulation, and minimally invasive precise positioning, which provides a guarantee for the clinician's decision-making judgment and helps the surgeon to accurately, quickly, and safely locate and insert the implant [12–15]. Therefore, robot-assisted orthopedic surgery is gradually widely accepted. However, there is no available evidence-based evidence to compare traditional cannulated screw internal fixation with computer-assisted percutaneous cannulated screw fixation on treating femoral neck fracture. For any emerging surgical technology and innovation, post-market assessment of its safety and efficacy is critical. It helps surgeons critically examine the advantages and limitations of adopting such technology in their practice [16, 17]. We carried out this meta-analysis to explore the clinical results of traditional manipulation and computer-assisted percutaneous cannulated screw fixation in treating femoral neck fracture.

## Methods

### Protocol and guidance

This study was performed by Preferred Reporting Items for Systematic Reviews and Meta-Analysis (PRISMA) [18]. The protocol for this review was registered with PROSPERO (CRD42020214493).

### Information sources and search strategy

We searched Embase, Pubmed, Web of Science, Cochrane Library, China National Knowledge Infrastructure (CNKI) and Wanfang Database from database inception to August 2020. We combined Medical Subject Headings (MSH) terms and free terms for searching, using the Pubmed search strategy as an example: ((((Femoral Neck Fracture[Title/Abstract]) OR (Femur Neck Fractures[Title/Abstract])) OR (Femur Neck Fracture[Title/Abstract])) OR ("Femoral Neck Fractures"[Mesh])) AND (((((((("Robotics"[Mesh]) OR ("Surgery, Computer-Assisted"[Mesh])) OR ("Robotic Surgical Procedures"[Mesh])) OR (Robotic Surgical Procedure[Title/Abstract])) OR (Robot-Enhanced Procedure*[Title/Abstract])) OR (Computer-Assisted Surger*[Title/Abstract])) OR (Computer-Aided Surger*[Title/Abstract])) OR (Image-Guided Surger*[Title/Abstract])).

### Inclusion criteria and exclusion criteria

The inclusion criteria were as follows:

(i) *Participants* All patients were definitively diagnosed with femoral neck fractures.
(ii) *Interventions* The experimental group was computer-assisted percutaneous cannulated screw fixation.
(iii) *Comparisons* The intervention for the control group was percutaneous cannulated screw fixation by traditional surgical methods.
(iv) *Outcomes* At least one of the following outcome indicators was reported: operation time, fluoroscopy frequency, intraoperative blood loss, intraoperative fluoroscopy times, Harris score, fracture healing rate, fracture healing time, and femoral head necrosis rate.
(v) *Study design* Randomized controlled trials (RCT), retrospective comparative control trial (CCT) and prospective cohort study (PCS) were included.

The exclusion criteria were as follows: repeated publications, case reports, letters, reviews, conference abstracts, study that unable to extract data, non-human and physical experimental studies, systematic reviews, and meta-analysis.

### Literature selection and data extraction

Two reviewers (Cheng and Lu) screened all the literature according to the inclusion and exclusion criteria [19]. Two reviewers independently extracted the following information: author, year of publication, study design, average age, sex, type of fracture and follow-up time, the results of quality evaluation, outcomes, and other general information. The primary outcome metrics included in the study were: operative time, Harris score, intraoperative bleeding volume, femoral head necrosis rate and fracture healing rate. Subgroup analysis was also performed on the main outcome indicators according to the type of computer-assisted equipment. Secondary outcome indicators included fracture healing time, fluoroscopy frequency and a total number of drills. Another investigator would resolve any disagreements.

### Risk of bias assessment

The risk of bias of RCTs was respectively assessed by two reviewers (Cheng and Lu) according to the Cochrane Collaboration tool [20]. The Cochrane Collaboration tool has seven domains: random sequence generation, allocation concealment, blinding of participants and personnel, blinding of outcome assessment, incomplete outcomes, selective outcome reporting, and other sources of bias. Each domain was classified as low, high, and unclear risk of bias. The quality of the non-randomized controlled trials (non-RCT) was assessed by the Newcastle–Ottawa scale (NOS) [21]. The assessment scale consisted of three domains: selection of study groups, comparability and exposure (case–control study) or outcome (cohort study). One star means one point, and the total score can reach nine points. The total score of 0–3, 4–6, and 7–9 can be divided into low quality, medium quality, and high quality.

### Statistical analysis

The meta-analysis was conducted by RevMan (Version 5.4). Standardized mean difference (SMD) and weighted mean differences (WMD), odds ratio (OR), and their corresponding 95% credible interval (95% CrI) were used to calculate continuous and dichotomous results, respectively. The *I*^*2*^ value and the Chi-square test was used to assess the heterogeneity; if the heterogeneity was low (*P* > 0.1, *I*^*2*^ ≤ 50%), a fixed-effects model was used. If the heterogeneity was high (*P* < 0.1*, I*^*2*^ > 50%), a random effect model was used. When the *P*-value was less than 0.05, it was considered to have statistical significance. In contrast, there was no difference in the data results between the two groups.

## Result

### Literature Screening

**Fig. 1:**
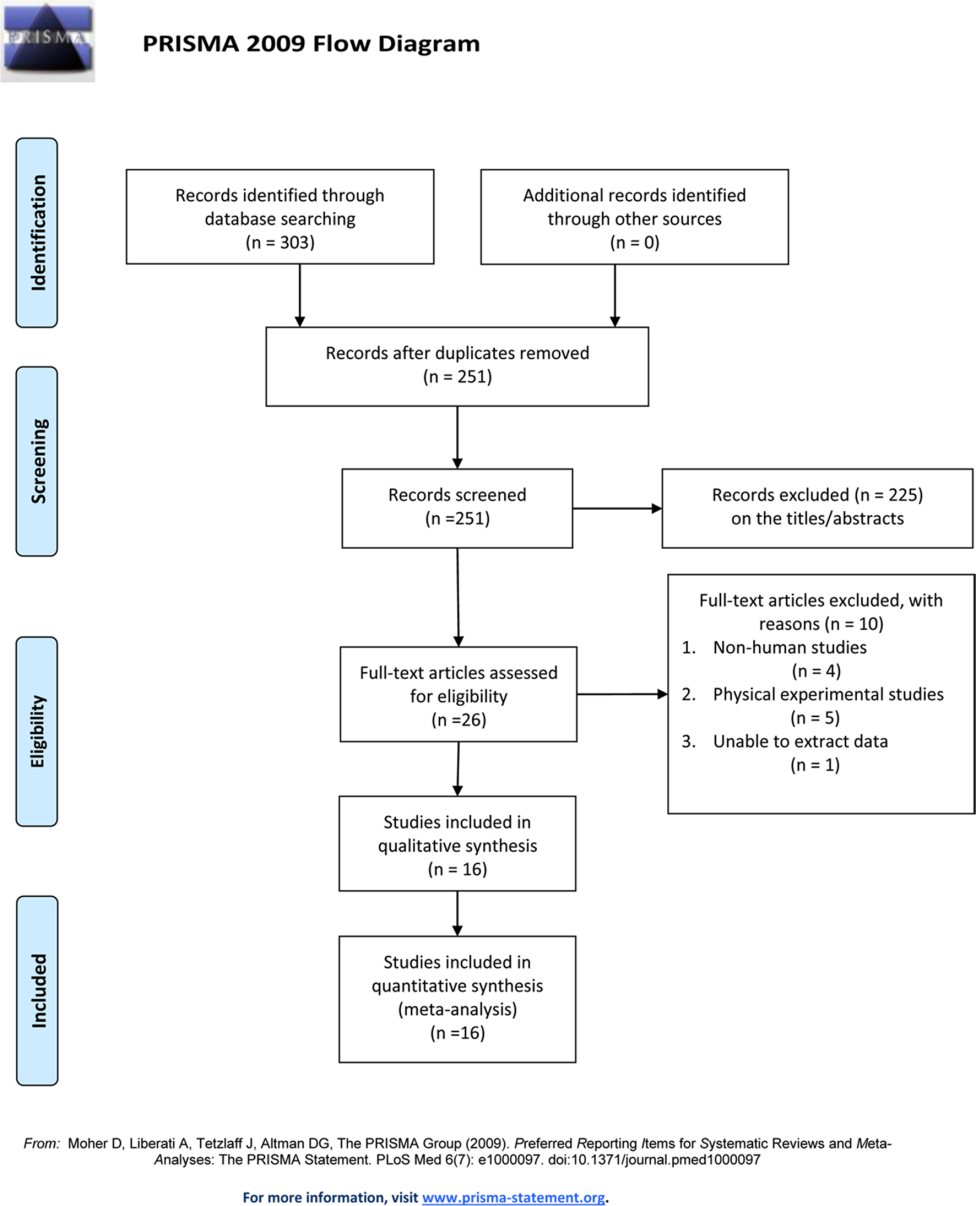
Preferred reporting items for systematic reviews and meta-analyses flow diagram of study selection

We searched six databases to retrieve a total of 303 studies, and 52 duplicate articles were removed using Endnote X9. The titles and abstracts were read to exclude 225 irrelevant studies. The full text of 26 articles was read carefully. Four articles were excluded due to non-human studies, five articles were excluded due to physical research experiments, and 1 article was excluded due to unavailable data extraction, resulting in 16 articles being included in this study. The information on the search process is provided in Fig. 1 (see Additional file 1). This study followed the PRISMA 2009 checklist as provided in Additional file 2.

### Study characteristics

A total of 1028 patients with femoral neck fractures were included in 16 studies published between 2006 and 2020, of which 493 patients were treated with computer-assisted methods, and 535 patients were treated with traditional methods. 12 studies [4, 10, 22–31] reported the following time, with the shortest being six months and the longest being 42 months. 10 studies [4, 22, 25–27, 30, 32–35] reported the operation time, 10 studies reported the Harris score, 8 studies [4, 10, 24, 25, 27–29, 34] reported the femoral head necrosis rate, five studies [4, 24–27] reported the fracture healing rate, and 10 studies[4, 23–27, 30–32, 34] reported the intraoperative bleeding. Secondary outcome indicators such as the number of drills, frequency of fluoroscopy, and fracture healing time were reported in 13 studies [4, 22–27, 30–35] 0.10 studies [4, 10, 23, 24, 26–28, 31, 34, 35] reported the type of computer-assisted device used, with five studies [4, 10, 23, 27, 31] used Ti-robot, two studies [35, 36] used GD-2000, one study [26] used Universal Robots, one used GD-A Robot, and one study [28] used iON FluroNav StealthStation navigation system. The study characteristics of these studies are shown in Table 1.

**Table 1:** General information of included studies

Study	Year	Study design	Number		Age (years)		Male/female			Follow-up (months)		Computer-assisted equipment
			Test group	Control group	Test group	Control group	Test group	Control group	Outcomes	Test group	Control group	
Lei 2019[20]	2019	RCT	44	44	42.1 ± 4.7	40.6 ± 5.3	24/20	22/22	A,F,G	12–24		Ti-robot
Huang 2017[21]	2017	PCS	32	32	59.4 ± 5.6	59.1 ± 4.9	10/22	12/20	B,E,F,G,H	19.4 ± 8.9	19.8 ± 8.0	–
Cao 2017[22]	2017	PCS	20	36	44.7	47.9	10/10	19/17	B,C,D,E,F,G	12–18		Universal Robots
Tong 2016[23]	2016	PCS	20	18	47.5	51.5	12/8	11/7	A,B,C,D,E,H	12–24		–
Ge 2016[24]	2016	PCS	13	25	59.0 ± 5.5	57.5 ± 4.8	8/5	11/14	A,B,D,E,F,H,	6–12		GD-A Robot
He 2019[10]	2019	CCT	30	30	56 (39–82)	56.2(80–34)	11/19	12/18	C	12–24		Ti-robot
Duan 2019[25]	2019	PCS	26	23	61.7 ± 5.2	62.1 ± 4.1	11/15	9/14	A,B,C,D,E,G,H	12–24		Ti-robot
Liebergall, M.2006[26]	2006	CCT	20	20	63.7 ± 14.5	70.6 ± 16.9	9/11	5/15	C	38(24–42)		iON FluroNav StealthStation navigation system
Wang 2019[4]	2019	PCS	63	65	49.03 ± 8.23	49.80 ± 7.68	30/33	31/34	A,B,C,D,E,F	12–24		Ti-robot
Li 2019[27]	2019	RCT	46	46	42 ± 4.7	40.6 ± 5.3	24/22	22/24	C	18		–
Liu 2014[31]	2014	CCT	17	19	45.1 ± 6.9	42.1 ± 7.7	7/10	8/11	A,F,G	–		–
Liu J 2015[32]	2015	CCT	21	25	65.2 ± 4.2	60.5 ± 5.1	8/13	11/14	B,C,E,G,H	–		GD-2000
Wen 2015[28]	2015	PCS	13	11	54.5 ± 7.3	51.3 ± 4.9	8/5	6/5	A,B,E,F	18(12–24)		–
Wu 2015[33]	2015	PCS	28	29	48	54	14/14	16/13	A,F,G	–		GD-2000
Yin 2020[29]	2020	PCS	28	29	48 (15–79)	54(22–80)	14/14	16/13	B,E,F	12–18		Ti-robot
Gan Z 2020[30]	2020	RCT	25	25	44.6 ± 1.8	46.8 ± 1.4	12/13	10/15	A,B,E,F,G	–		–

A, Operation time; B, Harris score; C, femoral head necrosis rates; CCT, retrospective comparative control trial; Control group, traditional method group; D, fracture healing rate; E, intraoperative bleeding volume; F, fluoroscopy frequency; G, total drilling times; H, fracture healing time; PCS, prospective cohort study; RCT, randomized controlled trials; Test group, computer-assisted group

### Quality assessment of the eligible studies

**Fig. 2:**
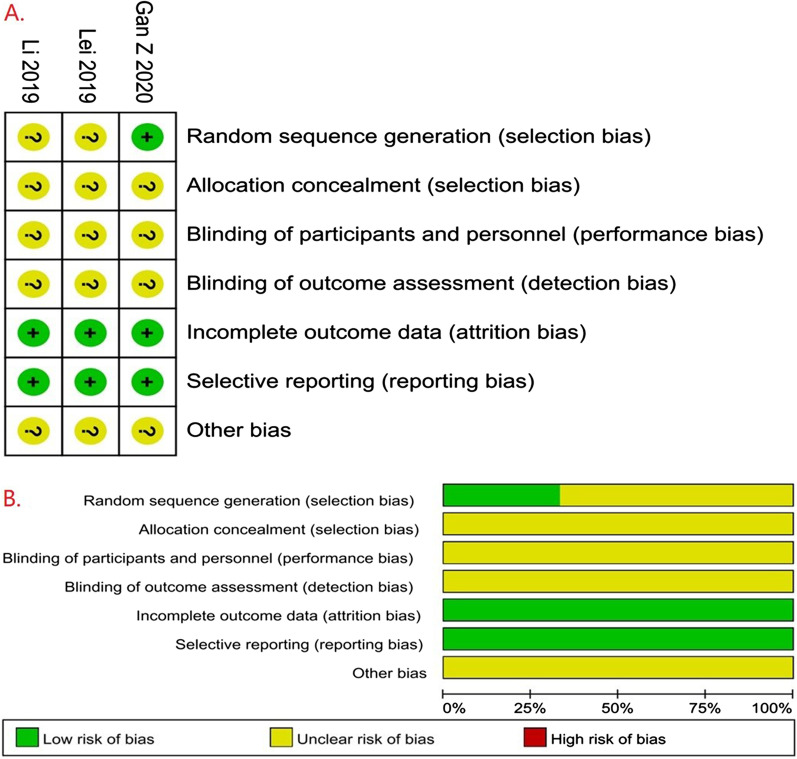
Risk of bias assessment of each included study: risk of **A** bias summary and **B** bias graph

A total of 16 studies were included, including three randomized controlled trials [22, 29, 32], nine prospective cohort studies [23–27, 30, 31, 35, 36], and five retrospective studies [4, 10, 28, 33, 34]. The risk of RCT bias was assessed by the Cochrane Collaboration Risk of bias tool. In the domain of sequence generation, two studies [22, 29] had an unclear risk of bias, one study [32] had a low risk of bias. In the domain of allocation concealment, blinding participants and personnel and terms of other biases, all studies [22, 29, 32] had an unclear risk of bias. In the domain of incomplete outcome data, all studies [22, 29, 32] had a low risk of bias. In terms of selective reporting, all studies [22, 29, 32] had a low risk of bias. Therefore, all studies were regarded as low quality (Fig. 2). The Newcastle–Ottawa scale assessed the quality of the non-RCT. Seven studies [4, 10, 23, 27, 30, 34, 35] scored six points, two studies [31, 33] scored seven points, and four [24–26, 28] studies scored five points(Table 2). Finally, twelve studies were regarded as medium quality and two studies were regarded as high quality.

**Table 2:** Quality assessment of non-randomized controlled trials (Newcastle–Ottawa scale for non-randomized controlled trials)

Study ID	Selection	Comparability	Exposure or Outcome	Total score
Ge 2016[24]	★★★		★★	5
Duan 2019[25]	★★	★★	★★	6
Cao 2017[22]	★★★		★★	5
Huang 2017[21]	★★★★		★★	6
Tong 2016[23]	★★★		★★	5
Liebergall. M 2006[26]	★★★		★★	5
He 2019[10]	★★★	★	★★	6
Wang 2019[4]	★★★	★	★★	6
Wen 2015[28]	★★★	★	★★	6
Wu 2015[33]	★★★	★	★★	6
Yin 2020[29]	★★★	★	★★★	7
Liu 2014[31]	★★★	★	★★★	7
Liu J 2015[32]	★★★	★	★★	6

### Meta-analysis and system review

#### Operation time (minutes)

Ten studies contained data referring to operation time, two of which were RCTs [22, 32] and eight of which were non-RCTs [4, 25–27, 30, 33–35], with a total of 519 patients. The overall heterogeneity between studies was high (*I*^*2*^ = 0% in RCTs, *I*^*2*^ = 79% in non-RCTs and *I*^*2*^ = 82% in overall 10 studies), and a random-effects model was used, and the results showed that the computer-assisted group had lower operating times than the traditional method group (MD = − 8.84, 95% CI: − 12.65, − 5.03; *P* < 0.00001).

**Fig. 3:**
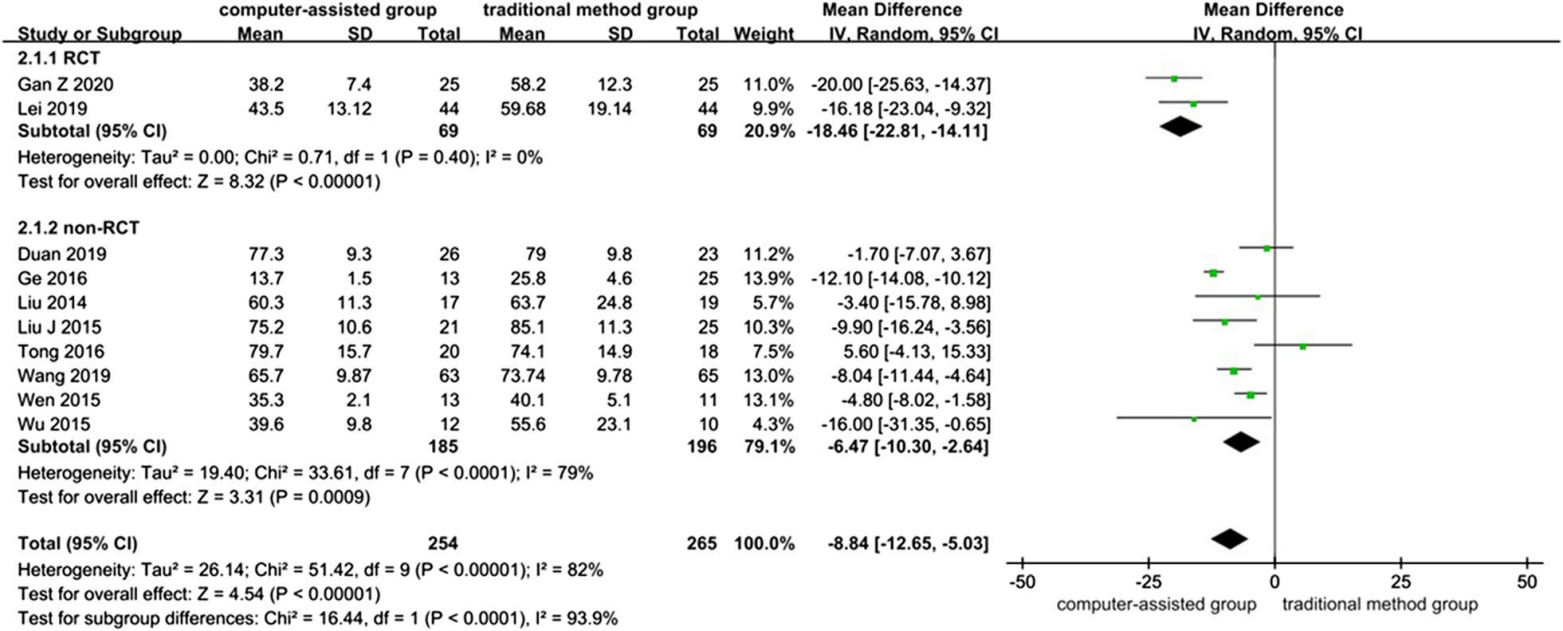
Forest plot diagram of compared operation time between traditional method group and computer-assisted group

**Fig. 4:**
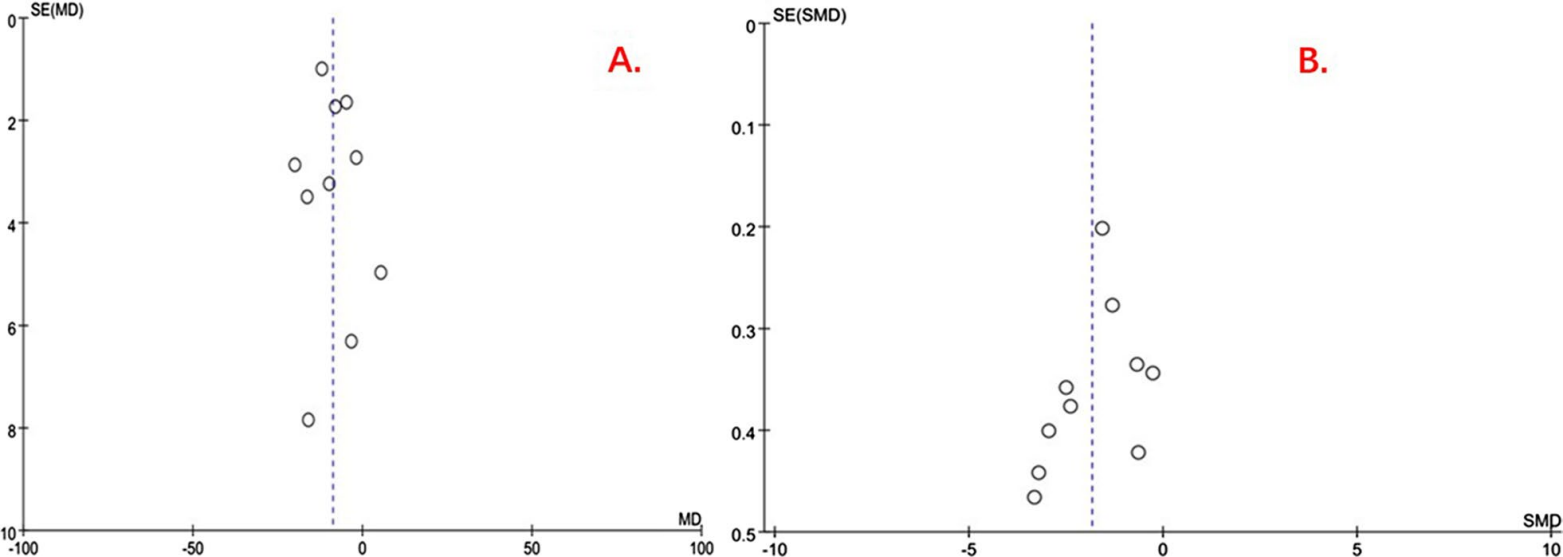
Funnel plot of **A** operation time and **B** intraoperative bleeding volume

The results of the RCTs showed that the computer-assisted group had significantly lower operating times compared to the traditional method group (MD = − 18.46, 95% CI: − 22.81, − 14.11; *P* < 0.00001), and the non-RCTs showed the same results (MD = − 6.47, 95% CI: − 10.30, − 2.64; *P* = 0.0009) (Fig. 3). The funnel plot is relatively symmetrical, indicating no possibility of publication bias (Fig. 4A).

### Harris score (points)

Ten studies contained data referring to Harris score, one of which was RCT [32] and nine of which were non-RCTs [4, 23–27, 30, 31, 34], with a total of 550 patients. The overall heterogeneity between studies was high (*I*^*2*^ = 64% in non-RCTs and *I*^*2*^ = 68% in overall 10 studies), and a random-effects model was used. The results showed that the Harris score was higher in the computer-assisted group than in the traditional method group (SMD = 0.69, 95% CI: 0.36, 1.01; *P* < 0.0001).

The systematic review of RCT showed that Harris scores were higher in the computer-assisted group than in the traditional method group *(P* < 0.0001).

**Fig. 5:**
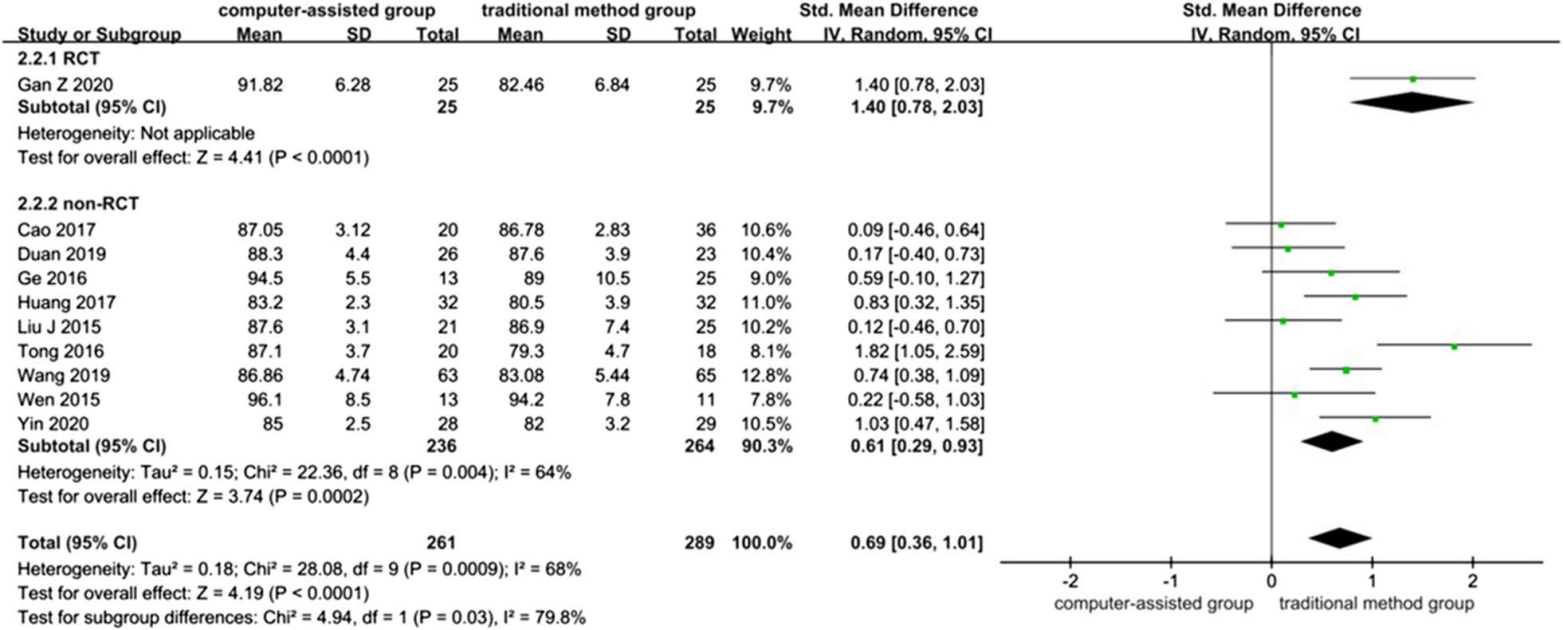
Forest plot diagram of compared Harris score between traditional method group and computer-assisted group

The meta-analysis of non-RCTs showed that the Harris score of the computer-assisted group was higher than the traditional method group. (SMD = 0.61, 95% CI: 0.29, 0.93; *P* = 0.0002) (Fig. 5).

### Femoral head necrosis rate

Eight studies contained data referring to femoral head necrosis rate, one of which was RCT [29], and seven were non-RCTs [4, 10, 24, 25, 27, 28, 34], with a total of 509 patients. Heterogeneity between studies was low (*I*^*2*^ = 0% in non-RCTs and *I*^2^ = 0% in overall 8 studies), and a fixed-effects model was used, which showed lower rates of femoral head necrosis in the computer-assisted group than in the traditional method group (OR = 0.36, 95% CI: 0.15, 0.85;* P* = 0.02).

The systematic review of RCT showed no significant difference in the rate of femoral head necrosis between the computer-assisted group and the group treated by traditional methods (*P* = 0.11).

**Fig. 6:**
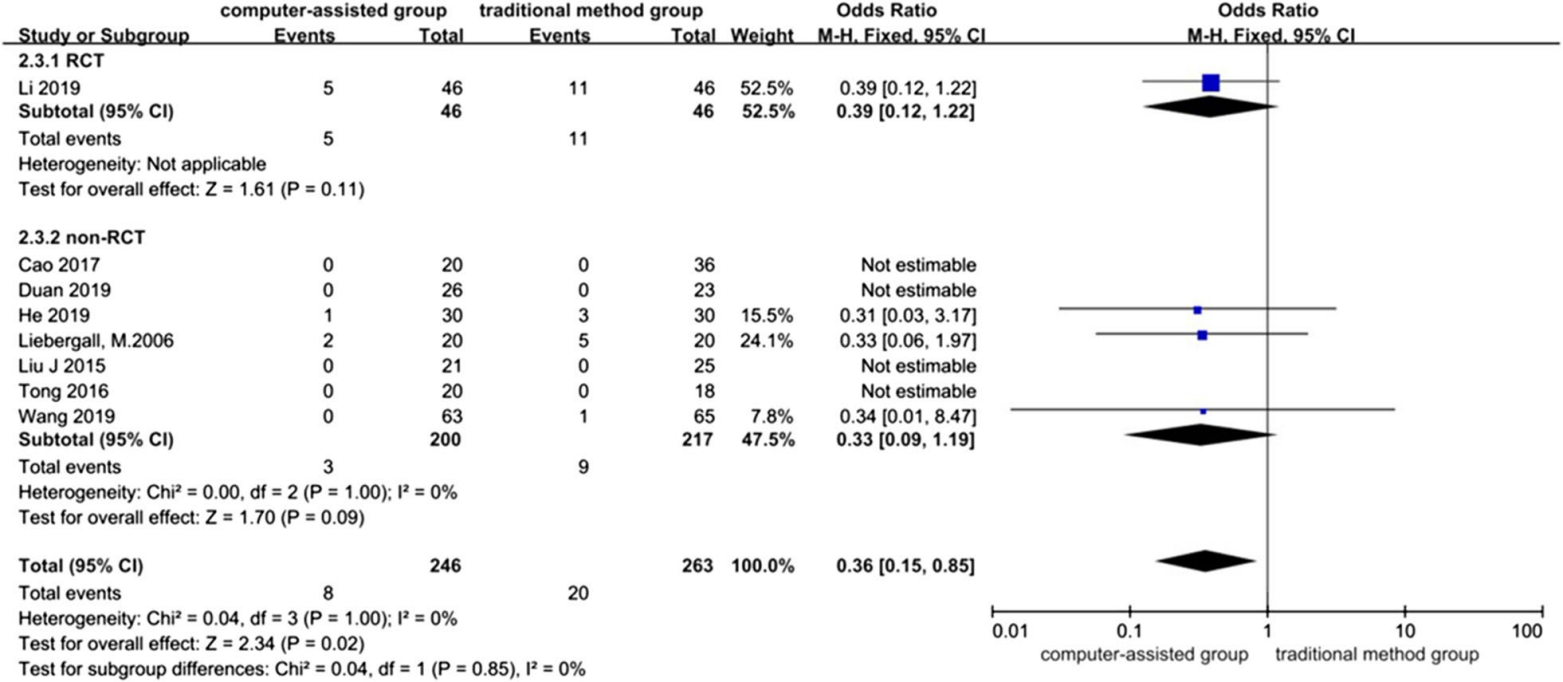
Forest plot diagram of compared femoral head necrosis rate between traditional method group and computer-assisted group

The meta-analysis of non-RCTs and results showed no significant difference in femoral head necrosis rate between the computer-assisted and conventional method groups (OR = 0.33, 95% CI: 0.09, 1.19; *P* = 0.09) (Fig. 6).

### Fracture healing rate

**Fig. 7:**
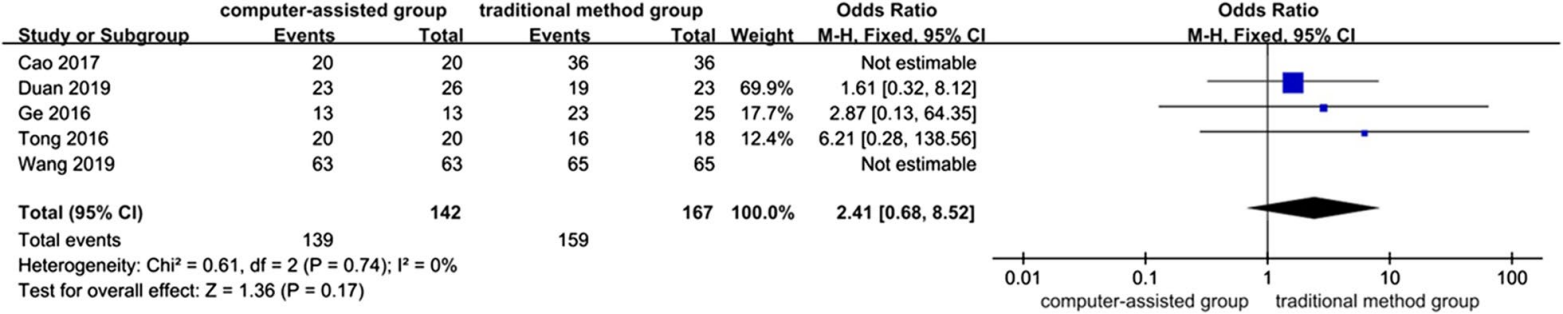
Forest plot diagram of compared fracture healing rate between traditional method group and computer-assisted group

The fracture healing rate of the computer-assisted group and traditional method group was compared. A total of 5 pieces of literature [4, 24–27] reported this result. A total of five articles mentioned fracture healing rate. All non-RCTs, because there was no significant heterogeneity between studies (*P* = 0.74, *I*^*2*^ = 0%). Analysis using a fixed-effects model showed no significant difference in fracture healing rate between the computer-assisted and conventional method groups (OR = 2.41, 95% CI: 0.68, 8.52; *P* = 0.17) (Fig. 7).

### Intraoperative Bleeding Volume (ml)

Ten studies contained data referring to intraoperative bleeding volume, one of which was RCT [32], and nine of which were non-RCTs [4, 23–27, 30, 31, 34], with a total of 550 patients. Overall heterogeneity between the studies was high (*I*^*2*^ = 89% in non-RCTs and *I*^2^ = 88% in overall 10 studies), and a random-effects model was used, which showed lower intraoperative bleeding volume in the computer-assisted group than in the traditional method group (SMD = − 1.84, 95% CI: − 2.46, − 1.22; *P* < 0.00001).

The systematic review of RCT showed that intraoperative bleeding was lower in the computerized group than in the conventional method group (*P* < 0.00001).

**Fig. 8:**
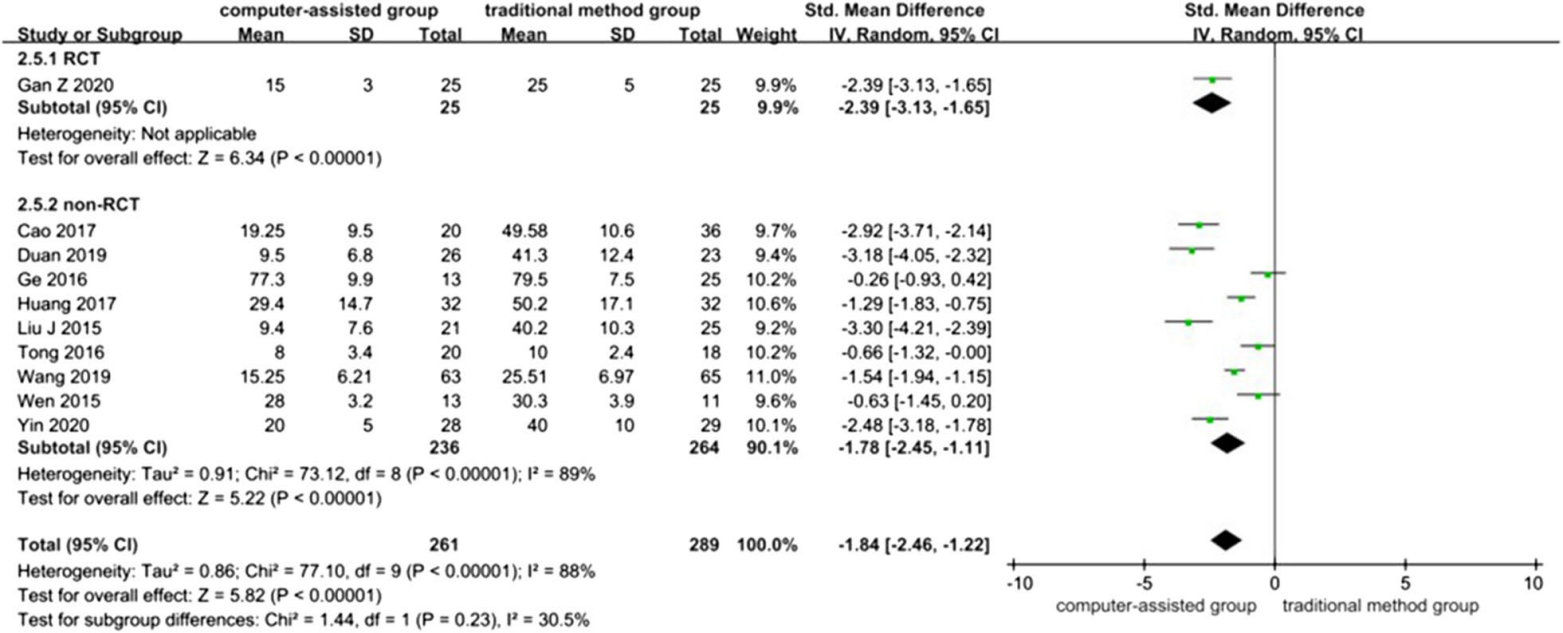
Forest plot diagram of compared intraoperative bleeding volume between traditional method group and computer-assisted group

The meta-analysis result of non-RCTs showed that intraoperative bleeding was lower in the computer-assisted group than in the traditional method group (SMD = − 1.78, 95% CI: − 2.45, − 1.11; *P* < 0.00001) (Fig. 8). The funnel plot is relatively symmetrical, indicating no possibility of publication bias (Fig. 4B).

### Meta-Analysis of secondary outcomes

Other secondary outcome indicators included fluoroscopy frequency, total drilling times, and Fracture healing time. Six non-RCTs [23–27, 34] reported fracture healing time, and the results showed no significant difference between the computer-assisted and conventional approach groups. The studies that reported the number of intraoperative fluoroscopies included two RCTs [22, 32] and ten non-RCTs [4, 23, 24, 26, 27, 30, 31, 33–35], and results showed that the number of intraoperative fluoroscopies was lower in the computer-assisted group than in the conventional method group. Two RCTs [22, 32] and six non-RCTs [23, 24, 27, 33–35] reported the total number of drills and showed a lower total number of drills in the computer-assisted group than in the conventional method group. The specific results are shown in Table 3.

**TABLE 3:** Secondary outcomes

Secondary outcomes	Type of research	Research number	Sample	Heterogeneity test		Statistic effect model	95%CI	*P*-value
				*P*-value	*I*^2^/%			
Fracture healing time (month)	RCT	–	–	–	–	–	–	–
	non-RCT	6(21–25,32)	291	0.59	0%	MD (Fixed, 95% CI)	− 0.24 [− 0.47, 0.00]	0.05
Total drilling times (number)	RCT	2(20,30)	138	0.0006	92%	SMD (Random, 95% CI)	− 6.13 [− 9.35,− 2.90	0.0002
	non− RCT	6(21,22,25,31–33)	274	< 0.00001	92%	SMD (Random, 95% CI)	− 3.09 [− 4.38,− 1.80]	< 0.00001
Fluoroscopy frequency (number)	RCT	2(20,30)	138	0.13	56%	SMD (Random, 95% CI)	− 6.64 [− 8.06,− 5.21]	< 0.00001
	non− RCT	10(4,21,22,24,25,28,29,31,33,34)	620	< 0.00001	91%	SMD (Random, 95% CI)	− 2.61 [− 3.40,− 1.82]	< 0.00001

*SMD* Std. mean difference, *MD* mean difference, *Random* random effects, *Fixed* fixed effects, *CI* confidence interval

### Subgroup analysis

We performed subgroup analyses of the operation time, intraoperative bleeding volume, and Harris score depending on the type of computer-assisted equipment because the specific type of computer-aided equipment is not specified in the RCTs. We only analyzed the non-RCTs, and the results are shown in Table 3.

### Sensitivity analysis

The primary outcome indicators, operation time, intraoperative bleeding and Harris score, had high heterogeneity, and sensitivity analysis was performed to determine the source of heterogeneity for these three outcome indicators.

**Fig. 9:**
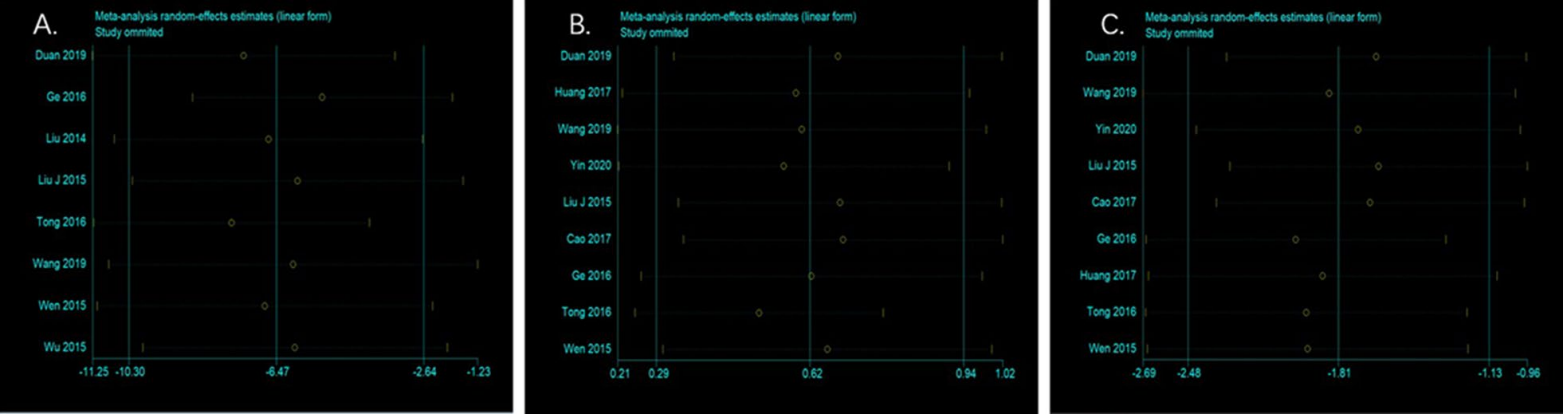
Sensitivity analysis of **A** operation time, **B** Harris score and **C** intraoperative bleeding volume

In the sensitivity analysis of operation time (Fig. 9A), the study of Ge et al. [26] and the study of Tong et al. [25] were found to be the main sources of heterogeneity. Their exclusion revealed a significant reduction in heterogeneity (*P* < 0.0001, *I*^*2*^ = 34%) and no directional change in the results of the statistical analysis (SMD = − 6.20, 95% CI: − 8.98, − 3.43; *P* < 0.0001). A careful reading of the two studies to find the reasons for the heterogeneity revealed that in Tong et al.'s study [25], the operation time in the computer-assisted group was higher than that in the conventional control group, which may be since the operators were not yet proficient in the use of computer-assisted equipment, resulting in their inefficient procedures and resulting in long operation times. In the study by Ge et al. [26], the computer-assisted and conventional method groups had significantly less operative time than the other studies due to different definitions of operative time. For example, only the operative time was recorded and did not include the time spent debugging the computer, resulting in an overall reduction in time.

In a sensitivity analysis of the Harris score (Fig. 9B), the study by Tong et al. [25] was found to be the main source of heterogeneity, and its exclusion revealed reduced heterogeneity (*P* = 0.09, *I*^*2*^ = 43%) and no directional change in the statistical analysis. A careful reading of this study revealed that patients in the computer-assisted group were younger than those in the traditional method group (47.5y vs 51.5y) and that younger patient was more likely to have higher Harris scores, which may account for the heterogeneity of the outcome, and the low quality of this study, which is considered a moderate quality study, may also be a source of heterogeneity.

No individual study showed significant heterogeneity in a sensitivity analysis of intraoperative bleeding (Fig. 9C). In a piecewise exclusion of the literature, no significant change in heterogeneity was found, suggesting that the results are robust.

## Discussion

Since the aging of the population has caused an increase in the number of patients with osteoporosis, which was the main cause of non-displaced femoral neck fractures in the elderly. At the same time, major car accident injuries and high-altitude fall injuries can also cause femoral neck fractures in young people. In the treatment of femoral neck fractures, percutaneous cannulated screw fixation was the first choice. Owing to its advantages of minimally invasive, less bleeding and reliable fixation, it has become a common internal fixation method for femoral neck fractures that involve a small displacement or for closed/limited open reduction [2, 37]. The application of computer-aided technology in orthopedic surgery has significantly improved the accuracy of the placement of femoral neck cannulated screws. Therefore, it has been accepted by more and more orthopedic surgeons [38]. This meta-analysis compared the clinical efficacy of computer-assisted hollow screw internal fixation with that of traditional manipulated hollow screw internal fixation for femoral neck fractures.

A total of 16 studies were finally included in our study. Among the main outcome indicators, operation time and intraoperative bleeding were important outcome indicators. Operation time was positively correlated with intraoperative bleeding, with longer operation time, longer anesthesia time and wound exposure time, and higher risk of intraoperative respiratory complications and intraoperative infections. As the duration of surgery continues to increase, the physician's stamina decreases and the likelihood of operational errors by the physician increases [39, 40]. During surgical operations, instruments capable of generating radiation are used to position implant, despite using protective clothing containing lead and lead plates during surgery, there is still a health risk to patients and surgeons due to prolonged operations [41, 42]. With computer-assisted hollow screw internal fixation for femoral neck fractures, the operation takes less time, and there is less intraoperative bleeding, which means that patients are less likely to have intraoperative complications, while the surgeon and the patient receive less radiation, thus protecting the health of the surgeon and the patient.

In the subgroup analysis, the results of the Ti-robot group, GD-2000 group and other group showed that the operation time and intraoperative bleeding were lower in the computer-assisted group than in the traditional method group. While some studies [25] demonstrated longer operative times in the computer-assisted group, the operative time decreases over time as proficiency in equipment operation increases [42]. The increase in the number of intraoperative drilling caused the risk of intraoperative infection and intraoperative bleeding. Multiple drilling leads to damage on the cortex and cancellous bone [2, 10, 17]. It may also lead to the risk of subtrochanteric fractures and postoperative complications [27]. The increase in the number of intraoperative drilling will inevitably lead to an increase in the number of intraoperative fluoroscopies [4]. Too much intraoperative fluoroscopy will have an adverse effect on the health of patients and doctors [4]. The results of our meta-analysis showed that the times of intraoperative drilling and intraoperative fluoroscopy in the computer-assisted group were less than those in the traditional method group.

Of the outcome indicators related to patient prognosis, the Harris score is a scale for evaluating hip function. Lan et al. [43] found that robot-assisted intramedullary nail fixation had a better Harris score for elderly patients with femoral intertrochanteric fractures compared with traditional surgical methods. In this study, the Harris score in the computer-assisted group was also higher than that in the traditional method group, indicating that the use of computer-assisted devices significantly improved the prognosis of patients. The results of the subgroup analysis showed that the use of the Ti-robot computing aid significantly improved patients' Harris scores, while the results of the GD-2000 and other groups showed no significant difference between the computer-assisted and traditional method groups, which may be because the Ti-robot was designed later compared to other computer-assisted devices and therefore was more advanced and had better features. Fracture healing rate and femoral head necrosis rate are also important prognostic indicators. Femoral head necrosis is an important postoperative complication of femoral neck fracture [44]. The hip replacement had to be performed after osteonecrosis of the femoral head [45]. Our results showed that the rate of femoral head necrosis was lower in the computer-assisted group than in the traditional method group. There was no significant difference in fracture healing rate between the two groups; this may be because the computer-assisted technique was used only as an adjunct and still relied on the operator's preoperative fracture repositioning and screw placement planning. Therefore, it resulted in similar follow-up results in both groups.

As the research on computational-assisted technology deepens, other advantages of computer-assisted technology are also discovered. With the assistance of a computer, percutaneous hollow nail fixation can make the robotic hand stable for a long time and avoid the fatigue of the surgeon holding the instrument for a long time, which has obvious advantages in the field of minimally invasive surgery and high-risk surgery, while achieving the best surgical results, the patient only suffers minor surgical injuries, which is more conducive to fracture healing and postoperative early rehabilitation exercises [4]. Also, a study by Leenders et al. [46] showed that the accuracy of experienced plastic surgeons performing surgical operations without computer navigation improved after using computer navigation in surgery.

Although current computer-assisted technology still has the following disadvantages: machinery and equipment are expensive, only a few people can afford the additional costs incurred in using computer-assisted equipment, and specialized learning and training are required [27, 47]. At the same time, for safety reasons, orthopedic surgery robots were usually used as auxiliary tools for surgery, and they cannot perform independent drilling operations [2]. However, with the continuous development of computer-assisted surgery in orthopedics in recent years, its functions will continue to improve. The computer-assisted motion compensation method for femoral neck fracture further improved computer-assisted femoral neck fracture accuracy and reduced the operation time and intraoperative blood loss [48]. Computer-assisted technology had obvious advantages in minimally invasive surgeries and high-risk surgeries. The current stage is only the initial stage of computer-assisted technology, and computer-assisted devices with more functions are constantly being developed. In the future, they can even be combined with artificial intelligence to perform fully automated screw implantation through deep learning, which will change the future surgery mode and the future surgery trend [47].

## Conclusion

In summary, computer-assisted surgery can overcome the shortcomings of traditional methods and improve the efficiency of surgery. It can also make doctors' operations safer, reduce X-ray injury of medical staff, and help patients have a better prognosis. Therefore, percutaneous cannulated screw fixation is a better choice for the treatment of femoral neck fracture. Besides, more centers, large samples and long-term follow-up randomized controlled trials are needed to provide stronger evidence for clinical use.

## The strengths and limitations

This study has the following strengths: 1) It is the first systematic review comparing computer-assisted percutaneous cannulated screw fixation for the treatment of femoral neck fractures and traditional cannulated screw internal fixation; 2) Two reviewers screened all the research literature based on inclusion and exclusion criteria. Two reviewers independently extracted the information to enhance the reliability of the research results.

This study has the following limitations: 1) The qualities of the included researches are generally low, and the number of high-quality RCT is relatively small. In more influential journals, the qualities of the reports are relatively better [49], but this study is relatively less influential; 2) most studies do not report complications in detail; 3) most of the included studies have a relatively small sample size, which reduces the credibility of the results; 4) most of the literature research areas included in this study are in China, and there is a lack of research literature from other countries; and 5) this study does not include the gray literature, which will exaggerate the estimation of the intervention effects and affect the study's final conclusion [50].

## Supplementary Information

Additional file 1: PRISMA flow diagram.

Additional file 2: PRISMA checklist.

## Data Availability

Data sharing is not applicable to this article as no datasets were generated or analyzed during the current study.

## Abbreviations

CNKI: China National Knowledge Infrastructure
MSH: Medical Subject Headings
RCT: Randomized controlled trials
CCT: Retrospective comparative control trial
PCS: Prospective cohort study
SMD: Standardized mean difference
WMD: Weighted mean differences
OR: Odds ratio
95% CI: 95% Confidence interval
NOS: The Newcastle–Ottawa Quality Assessment Scale
