# Supplementary material for: Computer-assisted cannulated screw internal fixation versus conventional cannulated screw internal fixation for femoral neck fractures: a systematic review and meta-analysis
Source: J Orthop Surg Res. 2021 Nov 22;16:687. doi: 10.1186/s13018-021-02806-7 (PMC8607593; doi:10.1186/s13018-021-02806-7)
Supplement: Supplementary file 1 — PRISMA flow diagram. [file 13018_2021_2806_MOESM1_ESM.doc]

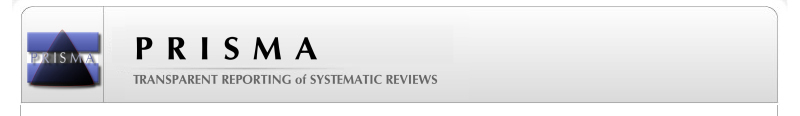
**PRISMA 2009 Flow Diagram**

**Screening**

**Included**

**Eligibility**

**Identification**

Records identified through database searching
(n =303)

Additional records identified through other sources
(n = 0)

Records after duplicates removed
(n =251)

Records screened
(n =251)

Records excluded (n = 225) on the titles/abstracts

Full-text articles assessed for eligibility
(n =26)

Full-text articles excluded, with reasons (n = 10)

1. No-human studies

(n=4)

1. Physical experimental studies

(n=5)

1. Unable to extract data

(n=1)

Studies included in qualitative synthesis
(n =16)

Studies included in quantitative synthesis (meta-analysis)
(n = 16)
